# Alteration of whole-brain amplitude of low-frequency fluctuation and degree centrality in patients with mild to moderate depression: A resting-state functional magnetic resonance imaging study

**DOI:** 10.3389/fpsyt.2022.1061359

**Published:** 2022-12-07

**Authors:** Fenyang Chen, Luoyu Wang, Zhongxiang Ding

**Affiliations:** ^1^The Fourth School of Clinical Medicine, Zhejiang Chinese Medical University, Hangzhou, Zhejiang, China; ^2^Department of Radiology, Affiliated Hangzhou First People’s Hospital, Zhejiang University School of Medicine, Hangzhou, Zhejiang, China

**Keywords:** mild to moderate depression, resting-state functional magnetic resonance imaging, low-frequency amplitude, degree centrality, receiver operator characteristic curve

## Abstract

**Background:**

Mild to moderate depressive disorder has a high risk of progressing to major depressive disorder.

**Methods:**

Low-frequency amplitude and degree centrality were calculated to compare 49 patients with mild to moderate depression and 21 matched healthy controls. Correlation analysis was conducted to explore the correlation between the amplitude of low-frequency fluctuation (ALFF) and the degree centrality (DC) of altered brain region and the scores of clinical scale. Receiver operating characteristic (ROC) curves were further analyzed to evaluate the predictive value of above altered ALFF and DC areas as image markers for mild to moderate depression.

**Results:**

Compared with healthy controls, patients with mild to moderate depression had lower ALFF values in the left precuneus and posterior cingulate gyrus [voxel *p* < 0.005, cluster *p* < 0.05, Gaussian random field correction (GRF) corrected] and lower DC values in the left insula (voxel *p* < 0.005, cluster *p* < 0.05, GRF corrected). There was a significant negative correlation between DC in the left insula and scale scores of Zung’s Depression Scale (ZungSDS), Beck Self-Rating Depression Scale (BDI), Toronto Alexithymia Scale (TAS26), and Ruminative Thinking Response Scale (RRS_SUM, RRS_REFLECTION, RRS_DEPR). Finally, ROC analysis showed that the ALFF of the left precuneus and posterior cingulate gyrus had a sensitivity of 61.9% and a specificity of 79.6%, and the DC of the left insula had a sensitivity of 81% and a specificity of 85.7% in differentiating mild to moderate depression from healthy controls.

**Conclusion:**

Intrinsic abnormality of the brain was mainly located in the precuneus and insular in patients with mild to moderate depression, which provides insight into potential neurological mechanisms.

## Introduction

Major Depressive Disorder (MDD)is a common and frequent psychiatric disorder mainly characterized by depressed mood, anhedonia, cognitive impairment, and somatic symptoms (1). Since the global rampage of COVID-19, the incidence of depression has been vastly increasing, with about 53.2 million new cases in 2020 alone (2). Hence, early identification of MDD has become an urgent topic in global mental health.

Scales crucial for the diagnosis of MDD include the International Classification of Disease-10 (ICD-10). Depression is divided into mild, moderate, and major depressive symptoms according to depression severity grading criteria. The diagnosis of major depressive symptoms including three typical symptoms and at least four other symptoms. Relative to major depressive symptoms, mild to moderate depressive symptoms have fewer core symptoms (3), which appear as non-specific symptoms and are likely to be mistaken for situational emotions or overlooked due to negative test results when atypical symptoms seek medical attention. Improper diagnosis and treatment may lead to serious adverse consequences. As the disease progresses, social function, and workability are progressively impaired, which will severely negatively impact one’s family and society. However, the most serious consequence of depression is suicide. According to statistics, depression is the leading cause of suicide and disability (4). In conclusion, it is important for early identification of major depression thus more timely clinical intervention can reach, such as simple and short term antidepressant therapy or behavioral cognitive intervention, which can reverse the progression to severe depression (5).

Amplitude of low-frequency fluctuation is an rs-fMRI-derived method to reflects the magnitude of spontaneous blood-oxygen-level-dependent (BOLD) signal (6). The amplitude of low-frequency fluctuations (ALFF), as a new marker of functional magnetic resonance imaging (fMRI), can reflect the intensity of regional neuronal activity (7). ALFF has been proven to have diagnostic significance in a variety of common psychiatric disorders, such as bipolar disorder (8), Alzheimer’s disease (9), schizophrenia (10), post-traumatic stress disorder (PTSD) (11), etc. Many studies have shown that depression is associated with abnormal activity in multiple brain regions. For example, Yang et al. (12) investigated ALFF values in depressed patients and found significantly lower levels in the left inferior parietal lobule and right caudate nucleus in untreated depressed patients. In addition, Gong et al. reported that ALFF values in the posterior cingulate gyrus, left inferior temporal gyrus, right superior temporal gyrus, right insula, right parietal lobe, and right fusiform gyrus were significantly increased in depressed patients. In contrast, ALFF values in the bilateral cuneus, left occipital lobe, and left medial frontal lobe were significantly decreased in depressed patients (13). There are differences results among different studies, probably owing to sample heterogeneity, various scanning equipment or parameters, preprocessing methods, or other treatment plans.

Degree centrality (DC) represents the number of direct connections for a given voxel in the voxel-based graphs. If a node is more connected to other nodes, the degree of centrality of the node will be higher. Compared with seed-based correlation analysis (SCA), DC does not require prior nodes or regions of interest and can capture the complexity of the functional connectome as a whole (14). A recent study reported by Zhou et al. (15) found abnormal DC values in the dorsolateral prefrontal cortex, insula, and posterior central gyrus in patients with first-episode non-medication MDD. Additionally, Li et al. (16) found that compared with healthy controls, the DC values of the inferior parietal lobule, parahippocampal gyrus, brainstem, and cerebellum in the late-onset depression group were significantly reduced. These results indicate that DC may help detect the underlying mechanism of depression. Although there are many studies on depression with ALFF, few studies on mild to moderate depression focus on ALFF or DC methods.

In this study, ALFF and DC were both used to detect the abnormal activity of brain function in patients with mild to moderate depression. We hypothesized that resting-state fMRI might be able to detect differences in specific brain regions between the mild to moderate depression group and the healthy control group.

## Materials and methods

### Subjects

Data obtained in the present study were acquired from the Open Database of Brain Imaging (OpenNEURO^1^). The Novosibirsk Tomography Center collected and provided rs-fMRI images, 3D-T1 images, and related clinical data. Inclusion criteria were as follows: Diagnosis was made according to the International Classification of Diseases (ICD-10) by an experienced psychotherapist; patients and healthy control aged 18–55 years old; not pregnant; not receiving anti-depressant treatment (and any psychotropic or cardiovascular function affecting medications); no significant psychiatric or neurological comorbidities; depression is non-seasonal and not secondary to any other condition; absence of contraindications to MR imaging; absence of rough structural brain abnormalities visible on T1 images. For mild depression (F32.0), the patients suffers from lowering of mood, lack of interest, or anhedonia, and at least two other symptoms, such as self-esteem and self-confidence reduced, some ideas of guilt or worthlessness and sleep disturbed and so on, which lasted for at least 2 weeks and did not affect social function. For moderate depression (F32.1), at least two of mild depression symptoms and at least four of the other symptoms should be present, lasting at least 2 weeks, and social functioning should be affected (See ICD-10 for more details).

Acceptable translation and rotation range <3 mm or 3°rotation. After excluding 2 cases that did not meet the diagnosis criteria of mild to moderate depression, 49 patients in the mild to moderate depression group and 21 healthy control group were finally enrolled [details of the open database can be found here^2^ (17) (18)].

### Image acquisition and data preprocessing

All subjects’ images were collected by the Philips 3.0T MRI system (Phillips, Netherlands). During scans, subjects were awake, eyes closed, head fixed, and lying quietly on the examination bed. Resting-state functional magnetic resonance image data was collected by echo plane imaging (EPI) sequence continuous scanning. Voxel size = 1.96 × 1.96 × 5 mm^3^, Repeat time (TR) = 2500 ms, echo time (TE) = 35 ms, Flip Angle = 90°, slice number = 25, slice thickness = 5 mm, field of view (FOV) = 220 × 220 mm^2^, matrix = 112 × 112, 100 volumes were collected. The voxel size of the structural image data is 0.87 × 0.87 × 1 mm^3^, TR = 7.5 ms, TE = 3.7 ms, flip angle = 8°, slice number = 181, slice thickness = 1 mm, FOV = 250 × 250 mm^2^, matrix = 288 × 288, slice gap = 0 mm. The present study used DPARSFA Toolbox (19) to preprocess the original data through the Matlab platform. The main steps are as follows: (1) Remove the first five volumes to obtain a stable scan (20); (2) Slice timing; (3) Head movement correction; (4) Registration of the structural image to functional image; (5) Spatial segmentation using 3D T1-weighted images; (6) Functional image data normalized to the Montreal Institute of Neuroscience (MNI) space, and re-sampled to 3 × 3 × 3 mm3 pixel size; (7) Covariables removed (including white matter, CSF signal, and 24 head parameters); (8) Bandpass filtering (0.01–0.08 Hz) performed on the time series of each voxel to remove the effects of low-frequency drift and high-frequency noise.

### Amplitude of low-frequency fluctuations

After preprocessing, a smoothing filter was used to reduce spatial noise and local anatomic artifacts; the full width at half maximum (FWHM) value of spatial smoothing was 4 mm. Then, Fast Fourier Transform (FFT) is applied to the filtered time series to transform the time domain signal to the frequency threshold to obtain the power spectrum. ALFF is calculated using the DPABI software by taking the square root of the power spectrum. For standardization purposes, the ALFF value of each voxel was divided by the global mean ALFF value (7).

### Degree centrality

After preprocessing, DC was also calculated using DPABI. DC takes each voxel as a node and calculates the correlation between this node and all other nodes in the whole brain. For nodes in binary graphs, DC is calculated by counting the number of edges connected. In contrast, DC is calculated in weighted graphs by calculating the sum of the weights of these edges. Compared with a binary graph, a weighted graph considers connection weights and can provide a more accurate representation of the centrality of the functional brain network (14). Generally speaking, the greater the degree of node centrality, the more vital it is in the network. The connection edge with correlation coefficient R >0.25 was selected as the default setting while constructing the DC map (21). At last, considering that smoothing may lead to overestimating local inter-voxel correlations, we put the smoothing step after DC calculation (22). The resulting matrices (DC maps) were smoothed with a Gaussian kernel (FWHM = 4 mm) to enable group comparisons and weighted graph calculation (23).

### Receiver operating characteristic curve of amplitude of low-frequency fluctuation and degree centrality calculation

We used receiver operating characteristic (ROC) curves to evaluate the predictive value of the abnormal ALFF and DC values of the above brain regions as imaging markers for mild to moderate depression and to explore the optimal value of ALFF and DC of the abnormal brain regions between mild to moderate depression patients and healthy controls. The area under the curve (AUC) and the sensitivity and specificity of the point value was calculated. *P* < 0.05 was considered statistically significant (24). ROC curves were analyzed and visualized using the ROCA toolkit based on Matlab.^3^

### Statistical analysis

SPSS 26.0 and DPABI were used for statistical analysis. First, SPSS 26.0 was used for statistical analysis of demographic and depression-related scales, and the Shapiro–Wilk test was used to test normality. The Chi-square test was used to compare gender differences between groups. The differences in ALFF and DC between the mild to moderate depression group and the healthy control group were analyzed using the DPABI two-sample *T*-test (voxel *P* < 0.005, cluster *P* < 0.05, *k* ≥ 25 voxels considered statistically significant). Multiple comparison analyses were corrected by Gaussian random field correction (GRF). Spearman correlation analysis was performed on the change values of each scale and brain functional imaging in all mild to moderate depression patients (*P* < 0.05 was considered statistically significant). Age and gender were used as covariables in group-level analysis.

## Results

### Comparison of demographic and clinical data

This research study recruited 49 patients and 21 healthy controls in total. There were no significant differences in age, sex, or IQ between two groups (*P* > 0.05; Table 1). The scores of Zung_SDS, BDI, TAS-26, ECR, RRS scales except MC-SDS scale were significantly higher than the scores of healthy controls (*p* < 0.05).

**TABLE 1:** Demographics and clinical characteristics.

Characteristic	Mild to moderate depression (*n* = 49)	Healthy controls (*n* = 21)	*t*/*X*^2^/*Z*	*P*-value
Age, mean (SD)	32.92 ± 8.98	33.81 ± 8.49	–0.387	0.70^a^
Sex (Male/Female)	11:38	6:15	–0.300	0.58^b^
IQ	103.43 ± 14.60	105.89 ± 16.47	–5.99	0.55^a^
Zung_SDS, mean (SD)	46.66 ± 7.12	32.67 ± 1.40	–5.207	0.00^c^
BDI, mean (SD)	20.87 ± 1.53	4.5 ± 1.09	–5.367	0.00^c^
MC-SDS, mean (SD)	11.48 ± 0.34	11.74 ± 0.53	–0.773	0.44^c^
TAS-26, mean (SD)	67.86 ± 1.76	54.72 ± 2.16	4.27	0.00^a^
ECR-avoid, mean (SD)	47.57 ± 2.11	36.56 ± 2.67	2.96	0.00^a^
ECR-and, mean (SD)	59.61 ± 2.57	49.28 ± 4.42	2.10	0.04^a^
RRS-sum, mean (SD)	55.44 ± 1.73	40.00 ± 2.37	5.015	0.00^a^
RRS-reflection, mean (SD)	12.56 ± 0.43	8.83 ± 0.67	4.73	0.00^a^
RRS-brooding, mean (SD)	11.88 ± 0.44	9,22 ± 0.66	3.31	0.00^a^
RRS-depression, mean (SD)	31.00 ± 1.02	21.94 ± 1.26	5.08	0.00^a^

^a^Two sample *t*-test; ^b^chi-square test; ^c^rank sum test; Zung_SDS, Zung’s Depression Scale; BDI, Beck Self-Rating Depression Scale; MC-SDS, Self-Rating Depression Scale; TAS-26, Toronto Alexithymia Scale; ECR, Experiences in Close Relationship; RRS, Ruminative Thinking Response Scale; IQ, intelligence quotient; SD: standard deviation.

### Amplitude of low-frequency fluctuation and degree centrality results

#### Amplitude of low-frequency fluctuation and degree centrality values of the mild to moderate depression group vs. healthy control group

**FIGURE 1:**
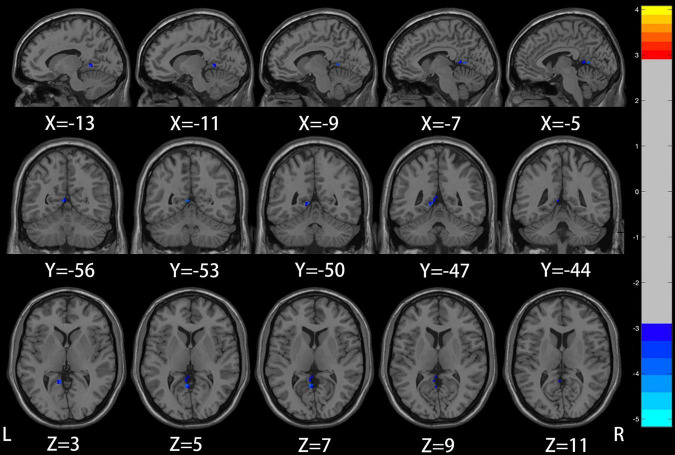
Compared to healthy controls, the region of reduced mALFF in mild to moderate depression patients was located in the left precuneus/posterior cingulate gyrus.

**FIGURE 2:**
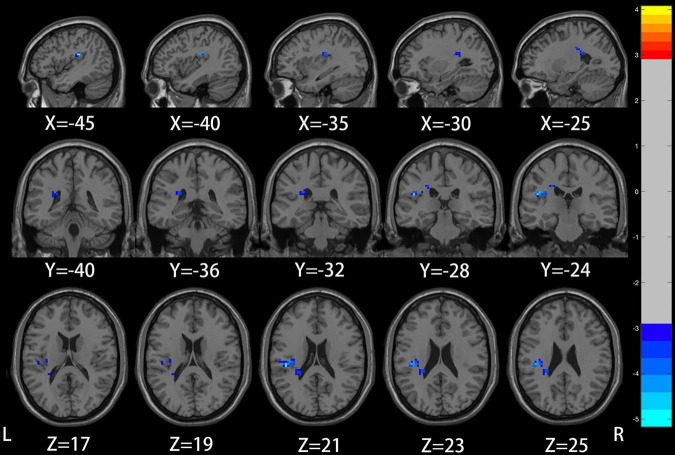
Compared to healthy controls, the region of reduced degree centrality (DC) in mild to moderate depression patients was located in the left insula.

Compared with the healthy control group, patients with mild to moderate depression had lower ALFF values in the left precuneus, posterior cingulate gyrus (voxel *P* < 0.005, cluster *P* < 0.05, GRF correction; Figure 1 and Table 2), and lower DC values in the left insula (voxel *P* < 0.005, cluster *P* < 0.05, GRF correction; Figure 2 and Table 3).

**TABLE 2:** Significant differences in ALFF between mild to moderate depression and healthy controls.

Brain regions (AAL)	Peak MNI coordinates (mm)			Cluster size	*T*-value
	X	Y	Z		
ALFF: Mild to moderate depression < HC					
PCC/PCu_L	−6	−54	6	17	−4.0331

Coordinates of the maximal point of the cluster and the associated *T*-values are shown in Montreal Institute of Neuroscience (MNI) spaces. After a two-sample *T*-test, the statistical significance threshold was set at voxel *P* < 0.005 and cluster *P* < 0.05 with false discovery rate Gaussian random field correction (GRF) correction. Age and gender were recorded as covariates. AAL, Anatomical Automatic Labeling; ALFF, amplitude of low-frequency fluctuation; PCC/PCu, posterior cingulate/precuneus.

**TABLE 3:** Significant differences in DC between mild to moderate depression and healthy controls.

Brain regions (AAL)	Peak MNI coordinates (mm)			Cluster size	*T*-value
	X	Y	Z		
DC: Mild to moderate depression < HC					
Insula_L	−45	−24	21	65	−5.1103

Coordinates of the maximal point of the cluster and the associated *T*-values are shown in Montreal Institute of Neuroscience (MNI) spaces. After a two-sample *T*-test, the statistical significance threshold was set at voxel *P* < 0.005 and cluster *P* < 0.05 with false discovery rate Gaussian random field correction (GRF) correction. Age and gender were recorded as covariates. DC, degree centrality.

#### Correlation analysis of amplitude of low-frequency fluctuation brain region changes and clinical scale

The ALFF values of the left precuneus/posterior cingulate gyrus did not conform to the normality test, and Spearman correlation analysis found no significant correlation with any scale.

#### Correlation analysis of degree centrality brain region changes and clinical scale

**FIGURE 3:**
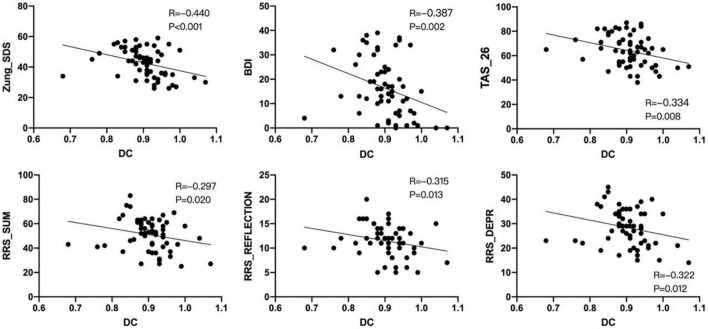
Non-parametric test–Spearman correlation analysis showed that the degree centrality (DC) signal of the left insula was negatively correlated with Zung_SDS, BDI, TAS_26, RRS_SUM, RRS_REFLECTION, and RRS_DEPR scores in the mild to moderate depression group (*P* < 0.05). ∙ Means the degree centrality of each subject in the left insula.

The DC values of the left insula did not match the normality test. And Spearman correlation analysis showed that the DC value of the left insula was significantly negatively correlated with the scores of Zung_SDS, BDI, TAS_26, RRS_SUM, RRS_REFLECTION, and RRS_DEPR scales in mild to moderate depression group (*p* < 0.05; Figure 3).

#### Receiver operating characteristic curve analysis

**FIGURE 4:**
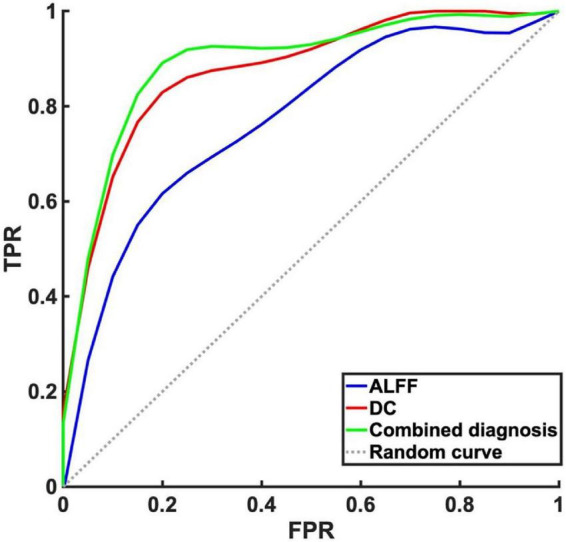
Receiver operating characteristic curve analysis of amplitude of low-frequency fluctuation (ALFF), degree centrality (DC) and combined diagnosis for altered brain regions. The area under the receiver operating characteristic (ROC) area under the curve (AUC) for ALFF in left precuneus/posterior cingulate gyrus was 0.77 (*P* < 0.001, 95% CI: 0.65–0.89), AUC for DC in left insula was 0.88 (*P* < 0.001, 95% CI: 0.79–0.96). Combined diagnosis for ALFF and DC was 0.89 (*P* < 0.001, 95% CI: 0.81–0.98).

There were significant differences in ALFF, DC values between mild to moderate depression and HC groups. We therefore assumed that the ALFF and DC could be applied to distinguish mild to moderate depression from healthy subjects. To test the hypothesis, ROC curves were used to analyze ALFF, DC values in distinct brain regions. The area under the ROC curve (AUC) represents the diagnostic rate. Values of 0.5–0.7 indicate low accuracy, 0.7–0.9 are middle accuracy, and >0.9 is high accuracy. The AUCs for ALFF and DC were 0.77, 0.88, respectively. Furthermore, we made a combination analysis of ALFF and DC with their altered brain areas, and the AUC was 0.89 (Figure 4 and Table 4). Therefore, these results may help to diagnose mild to moderate depression.

**TABLE 4:** ROC and AUC of amplitude of low-frequency fluctuation (ALFF), degree centrality (DC).

Method	Group	AUC	*P*-value	Sensitivity	Specificity	95% CI
ALFF	Mild to moderate depression vs. HC	0.769	<0.001	61.9%	79.6%	65–89%
DC	Mild to moderate depression vs. HC	0.875	<0.001	81.0%	85.7%	79–96%
ALFF + DC	Mild to moderate depression vs. HC	0.894	<0.001	85.7%	87.8%	81–98%

ROC, receiver operating characteristic; AUC, area under the ROC curve; HC, healthy control; CI, confidence interval.

## Discussion

To our knowledge, this is the first study to investigate functional changes in brain regions based on two different rsfMRI metrics in patients with mild to moderate depression, including ALFF and DC. Image analysis between the groups showed that compared with healthy control group, spontaneous low-frequency oscillation abnormities and degree centrality both found abnormal brain regions in the mild to moderate depression group, located in the precuneus, posterior cingulate gyrus (PCu/PCC) and insula. Moreover, we find DC value of the left insula was significantly negatively correlated with the scores of Zung_SDS, BDI, TAS_26, RRS_SUM, RRS_REFLECTION, and RRS_DEPR scales in mild to moderate depression group. The ROC analysis also showed that the ALFF, DC of above abnormal areas and combination of both metrics had relatively high sensitivity and specificity, which could effectively in differentiating mild to moderate depression from healthy controls.

One of the core symptoms of depression include overgeneralization of autobiographical memory, a manifestation of cognitive impairment that is associated with depression’s avoidance of recalling specific memories (25). Evidence shows that the precuneus is vital in integrating psychological processing into cognitive control processes, such as visual intention, episodic memory, and self-directed operation (26). The precuneus is also one of the critical nodes in the default mode network, and its abnormality will lead to cognitive impairment and emotional abnormalities (27, 28). In addition, the posteromedial region of the default network in the resting state is related to autobiographical memory, which is characterized by over-generalization and a vague memory of particular adverse events in patients with depression, which may be a defense mechanism to reduce painful emotional experiences when recalling specific adverse events (27). A study using ALFF, fALFF, and ReHo in first-episode untreated depression showed significantly lower precuneus values than healthy controls (29). Another study using dALFF and dFC in major depressive disorder, bipolar disorder and healthy controls, found both MDD and BD groups have significantly decreased temporal variability of the dALFF (less dynamic segregation) in bilateral PCC/PCu compared with the HCs (30). In addition, the function of precuneus in patients with mild to moderate depression has not been reported. Hence, mechanistic abnormalities associated with the precuneus remain to be elucidated. Our study found that patients with mild to moderate depression showed lower activity in the precuneus region, which is consistent with MDD study (29). ALFF is considered to reflect the extent of spontaneous neuronal activity (SNA), and decreased ALFF value indicates decreased oxygen consumption in specific brain areas (7). However, we observed that the number and range of abnormal brain areas in the patients in this study were less than those in the studies of MDD. We speculate that it might because the clinical symptoms of mild to moderate depression are less severe than MDD patients.

The limbic system plays a vital role in the formation of depression, and the insula is an integral part of the limbic system. The insula is believed to be related to human emotion and consciousness and plays a role in processing sensation, cognitive function, self-awareness, and complex social functions. At the same time, the insula is a crucial node region that initiates network exchange in significant networks, and the structure and function of the insula are often changed in depression (31). The present study found that compared with the healthy control group, the DC value of the left insula decreased, which was consistent with the results of Gao et al. (32). DC is a data-driven method based on Eigenvector Centrality Mapping (ECM), which can accurately and objectively detect all brain regions as communication hubs (33). We observed decreased DC values in the left insula, suggesting that this node may be important for patients with mild to moderate depression. Therefore, we hypothesized that, similar to patients with MDD, abnormal insula outcomes would be important for patients with mild to moderate depression. In addition, although only insula abnormalities were observed in this study, correlation analysis showed that left insula DC values were negatively correlated with Zung_SDS, BDI Self-Rating Depression Scale, Toronto Alexithymia Scale, and Ruminant Response Scale in the mild to moderate depression. It is suggested that the severer the extent of limbic system damage, the severer the symptoms of depressive symptoms, alexithymia and rumination would be. Moreover, ROC curves were plotted for individual and combined measures for two RS-fMRI measures showed differences between mild to moderate depression and healthy controls. The results showed that the combined measurement model based on ALFF and DC could better predict the probability of mild to moderate depression.

The present study has some limitations. First of all, the sample size of healthy control group should be expanded in future studies. Secondly, a major depressive disorder group should be added for comparing with healthy controls for a more comprehensive analysis. Lastly, this is a cross-sectional study; future studies are needed to confirm longitudinal brain activity changes.

In the present study, we compared the brain function imaging performance of mild to moderate depression group and healthy control group on two metrics: local brain indicator ALFF and whole brain network indicator DC. We identified regional spontaneous brain activity abnormalities in several brain regions in mild to moderate depression. These abnormal brain regions have similarities with the impaired patterns in MDD individuals. Particularly, abnormal network properties in the left insula, were associated with higher level of depression traits, indicating that rs-fMRI abnormalities in this area could be more likely to reflect the neurobiological feature of mild to moderate depression. Moreover, the combined application of rs-fMRI metrics can effectively predict the possibility of mild to moderate depression, which could has a good potential for clinical application.

## Data availability statement

The datasets presented in this study can be found in online repositories. The names of the repository/repositories and accession number(s) can be found below: https://openneuro.org/datasets/ds002748/versions/1.0.5.

## Ethics statement

The studies involving human participants were reviewed and approved by local ERB of Research Institute of Molecular Biology and Biophysics (which is currently a division of Federal Research Center of Fundamental and Translational Medicine, Novosibirsk, Russia), reference No. 1 from 8 June, 2016. The patients/participants provided their written informed consent to participate in this study. Written informed consent was obtained from the individual(s) for the publication of any potentially identifiable images or data included in this article.

## Author contributions

FC proposed the idea, conducted experiments and data processing, and wrote the manuscript. LW provided important help on guiding the experiments and analysis methods. ZD revised the manuscript and gave important advices. All authors contributed to the article and approved the submitted version.

## Footnotes

1
http://openneuro.org

2
https://openneuro.org/datasets/ds002748/versions/1.0.5

3
https://github.com/Luoyu-Wang/ROCA
