# Supplementary material for: Alteration of whole-brain amplitude of low-frequency fluctuation and degree centrality in patients with mild to moderate depression: A resting-state functional magnetic resonance imaging study
Source: Front Psychiatry. 2022 Dec 7;13:1061359. doi: 10.3389/fpsyt.2022.1061359 (PMC9768018; doi:10.3389/fpsyt.2022.1061359)
Supplement: Supplementary file 1 [file Data_Sheet_1.PDF]

Data supplement for Chen et al., Alteration of whole-brain ALFF and degree centrality in patients with mild to moderate depression: A resting-state fMRI study

CONTENTS

Appendix 1. Details of Assessment scale

1. Zung's Depression Scale:
   The Zung's Self-Rating Depression Scale (ZungSDS) was published by Zhong's in 1965. It is a simple and convenient self-rating scale for depression, including 20 items, which can directly reflect the subjective feelings of depressed patients, provide preliminary screening and early assessment for depression.
2. Beck Self-Rating Depression Scale(BDI):
   Beck Self-Rating Depression Scale is one of the most widely used measures for assessing the severity of depression in psychiatric patients and screening for possible depression in normal populations. Relative to BDI-I, BDI-II was modifyed to make it more consonant with DSM-III/IV criteria, and it was specifically constructed to measurethe severity of self-deported depression in adolescents and adults according to DSM-IV criteria for diagnosing depressive disorders.
3. Toronto Alexithymia Scale (TAS26):
   Alexithymia, characterized by an inability to properly express emotions and a lack of fantasy and practical thinking. It can be a negative personality trait, or a psychological characteristic commonly seen in some physical or mental diseases, or a secondary symptom. TAS26 has high reliability and validity, which can comprehensively and correctly evaluate the existence and severity of alexithymia, and take different treatment measures for the disease.
4. Ruminative Thinking Response Scale (RRS_SUM, RRS_REFLECTION, RRS_DEPR):
   Ruminative thinking response scale was published by Nolen-Hoeksema, which includes 22 items, and factor structure and psychometric specificity of RRS was composed of Brooding, Reflection and Depression proposed by Treynor et al. RRS has been proved to have high reliability and validity, which has been widely used in clinical and normal groups of all ages.
